# *n*-3 PUFA-Enriched Diet Preserves Skeletal Muscle Mitochondrial Function and Redox State and Prevents Muscle Mass Loss in Mice with Chronic Heart Failure

**DOI:** 10.3390/nu15143108

**Published:** 2023-07-11

**Authors:** Gianluca Gortan Cappellari, Aneta Aleksova, Matteo Dal Ferro, Antonio Cannatà, Annamaria Semolic, Alberto Guarnaccia, Michela Zanetti, Mauro Giacca, Gianfranco Sinagra, Rocco Barazzoni

**Affiliations:** 1 Department of Medical, Surgical and Health Sciences, University of Trieste, 34149 Trieste, Italy; 2 Cattinara Hospital, Azienda Sanitaria Universitaria Giuliano Isontina (ASUGI), 34149 Trieste, Italy; 3 Cardiothoracovascular Department, Azienda Sanitaria Universitaria Giuliano Isontina (ASUGI), 34128 Trieste, Italy; 4 School of Cardiovascular and Metabolic Medicine & Sciences, King’s College London, London WC2R 2LS, UK; 5 Molecular Medicine Laboratory, International Centre for Genetic, Engineering and Biotechnology (ICGEB), 34149 Trieste, Italy

**Keywords:** *n*-3 PUFA, skeletal muscle, chronic heart failure, muscle wasting

## Abstract

**Rationale and Methods:** Skeletal muscle derangements, potentially including mitochondrial dysfunction with altered mitochondrial dynamics and high reactive oxygen species (ROS) generation, may lead to protein catabolism and muscle wasting, resulting in low exercise capacity and reduced survival in chronic heart failure (CHF). We hypothesized that 8-week *n*-3-PUFA isocaloric partial dietary replacement (Fat = 5.5% total cal; EPA + DHA = 27% total fat) normalizes gastrocnemius muscle (GM) mitochondrial dynamics regulators, mitochondrial and tissue pro-oxidative changes, and catabolic derangements, resulting in preserved GM mass in rodent CHF [Myocardial infarction (MI)-induced CHF by coronary artery ligation, left-ventricular ejection fraction <50%]. **Results:** Compared to control animals (Sham), CHF had a higher GM mitochondrial fission-fusion protein ratio, with low ATP and high ROS production, pro-inflammatory changes, and low insulin signalling. *n*-3-PUFA normalized all mitochondrial derangements and the pro-oxidative state (oxidized to total glutathione ratio), associated with normalized GM cytokine profile, and enhanced muscle-anabolic insulin signalling and prevention of CHF-induced GM weight loss (all *p* < 0.05 vs. CHF and *p* = NS vs. S). **Conclusions:** *n*-3-PUFA isocaloric partial dietary replacement for 8 weeks normalizes CHF-induced derangements of muscle mitochondrial dynamics regulators, ROS production and function. *n*-3-PUFA mitochondrial effects result in preserved skeletal muscle mass, with potential to improve major patient outcomes in clinical settings.

## 1. Introduction

Loss of skeletal muscle mass often occurs in chronic heart failure (CHF) with a strong negative impact on patient outcomes [1,2], at least partly independent of myocardial dysfunction [3,4,5,6]. Low skeletal muscle mass may negatively affect exercise capacity [4,5], fitness [4,7], and ultimately survival [7,8], and the preservation of muscle mass is, therefore, an emerging relevant therapeutic target in CHF. Skeletal muscle mitochondrial derangements may contribute to skeletal muscle wasting in CHF, as well as other chronic diseases and aging [9,10,11,12,13]. We recently described enhanced skeletal muscle mitochondrial reactive oxygen species generation (ROS) with tissue oxidative stress and impaired ATP production and oxidative capacity in rodent CHF models with low ejection fraction [14,15], notably consistent with clinical reports [11]. In rodent models, mitochondrial derangements were associated with clustered tissue catabolic abnormalities including pro-inflammatory cytokine changes and impaired insulin signalling, leading to low skeletal muscle mass [14,16,17,18]. Recent studies [19,20,21] further demonstrated that altered regulation of mitochondrial dynamics with higher mitochondrial fission-fusion protein expression may causally contribute to mitochondrial dysfunction and pro-oxidative derangements in chronic disease conditions, by altering mitochondrial morphology and quality through impaired mitophagy [22,23].

*n*-3 polyunsaturated fatty-acids (*n*-3 PUFA) are emerging regulators of mitochondrial function in different cell types and tissues in experimental models [24,25,26]. In non-muscle cells, *n*-3 PUFA were notably reported to enhance the expression of master regulators of mitochondrial biogenesis [27,28,29], and to exert anti-oxidative activities in physiological conditions, as well as in obesity and healthy aging [28,30,31]. *n*-3 PUFA were also reported to enhance mitochondrial fusion proteins in non-muscle tissues in obesity [32]. Importantly, we recently comprehensively investigated the impact of *n*-3 PUFA on a cluster of skeletal muscle mitochondrial and catabolic pathways in a 5/6 nephrectomy-induced rodent model of chronic kidney disease (CKD) [19]. In the above study, *n*-3 PUFA isocaloric dietary replacement for one month normalized CKD-induced derangements in mitochondrial dynamics, ROS production, and oxidative capacity, with preserved tissue anabolic signalling and muscle mass [19]. Low-dose long-term *n*-3-PUFA supplementation has been previously investigated in patients with CHF based on anti-inflammatory and anti-arrhythmic activities, resulting in significantly lower mortality [33,34], with no information on skeletal muscle-related variables. Here, we therefore tested the hypothesis that *n*-3 PUFA isocaloric partial dietary replacement prevents skeletal muscle catabolism and loss of muscle mass in low-ejection fraction rodent CHF, with normalization of mitochondrial dynamics regulators and prevention of mitochondrial dysfunction and tissue pro-oxidative changes. CHF was triggered by coronary artery ligation, resulting in myocardial infarction and decreased left ventricular ejection fraction (LVEF), as previously reported [14], in 10-weeks-old mice.

## 2. Methods

**Experimental protocol**—Thirty 10-week-old male CD1 mice (Harlan, San Piero al Natisone, Italy) were used for the current study, which was conducted in compliance with national and international laws and policies. The ICGEB Animal Welfare Board and Ethical Committee and the National Animal Experimentation Authority (decree ref. 6442015PR) reviewed and approved the study design and procedures. Animals were housed in the International Centre for Genetic Engineering and Biotechnology (ICGEB, Trieste, Italy) animal facility at constant temperature and humidity and exposed to 12 h light/dark cycles with ad libitum access to water and food at all times. Body weight and food intake were monitored twice a week. Weight difference between the amount of chow provided at the start and that remaining at the end of each measurement was used to calculate daily energy intake, according to chow caloric content as reported by the manufacturer. Heart failure was induced in 20 randomly chosen animals by permanent left anterior descending (LAD) coronary artery ligation (CHF), as previously described [14]. The remaining ten animals underwent sham operation (S). To further evaluate cardiac function and confirm CHF, 2-D transthoracic echocardiography was performed using a Vevo 770 Ultrasound device (Visualsonics, Bothell, WA, USA) as previously reported [14], both after surgery and one week before sacrifice. Animals who underwent LAD ligature but whose LVEF was not reduced to <50% were excluded from the study, leaving n = 8 animals/group. Ecocardiography also confirmed that all sham-operated animals had LVEF >50%. Animals with suitable LVEF were then randomly assigned to standard diet (CHF, n = 10: 18% total calories from fat, 58% carbohydrates, 24% proteins; Teklad global diet 2018, 3.1 kcal/g, Harlan) or modified PUFA-enriched isocaloric isolipidic diet (CHF-PUFA, n = 10) obtained by replacement of soybean-derived fat with highly refined *n*-3 PUFA preparation (27% of total dietary lipids; Harlan, Italy; *n*-3 PUFA preparation was 600 mg/g in triglycerides formulation by EPAX, Ålesund, Norway), with unchanged content for other macro- and micro-nutrients [19,35]. Sham-operated animals were kept on standard diet. After 8 weeks dietary treatment (i.e., 56 days after surgery), anesthesia (thiobutabarbital 100 mg/kg, tiletamine/zolazepam [1:1] 40 mg/kg, i.p.) was performed after 3-h fasting, followed by immediate surgical isolation and removal of gastrocnemius muscles. Left gastrocnemius was cleaned and weighed on a technical scale, and tissue aliquots were prepared and placed in iced saline for quick transfer to ex vivo processing or snap frozen for other analyses [36]. Blood samples were then collected by heart puncture, followed by plasma separation by centrifugation and storage at −80 °C. Plasma glucose concentration was assessed by standard enzymatic-colorimetric assay [37].

**Mitochondrial enzyme activity and ex vivo ATP synthesis**—Mitochondrial citrate synthase (CS) enzyme activity was measured kinetically in muscle homogenates by spectrophotometry, as reported by [15]. Briefly, sample CS–induced coenzyme A (CoA) regeneration during incubation with acetyl-CoA (0.25 mM) and oxaloacetate (7.6 mM) is detected by subsequent conversion of 5′, 5′-Dithiobis 2-nitrobenzoic acid (DTNB; 0.025 mM) to 2-nitrobenzoic acid, a coloured (412 nm) dye, over time. For ATP synthesis assessment, mitochondria were isolated in homogenized fresh tissue by differential centrifugation. After a first separation (720× *g*, 15 min, 4 °C) in which nuclei and debris were removed, mitochondria were pelleted (10,000× *g*, 15 min, 4 °C) and resuspended. The luciferin-luciferase luminometric assay (ATP Reagent SL, BioThema, Handen, Sweden) was used to assess ATP synthesis rate, by kinetic assessment of light emission (interval 2 min, integration time 1 s) using a microplate luminometer (Synergy 2 SL, BioTek, Winooski, VT, USA) with different combinations of respiratory substrates (concentrations (mmol/L): 0.25 pyruvate, 0.0125 palmitoyl-L-carnitine, 2.5 α-ketoglutarate, 0.25 malate (PPKM); 0.025 palmitoyl-L-carnitine, 0.5 malate (PCM); 20 succinate, 0.1 rotenone (SR); 10 glutamate, 5 malate (GM)) [15,36,37]. In particular, ATP synthesis rate was measured on the linear phase after ADP (100 µM) addition, and a standard ATP curve was used to interpolate light emission. CS activity was used to normalize results, which were expressed as µmol (U CS)^−1^ min^−1^.

**Protein analyses**—Protein levels of mitochondrial dynamin like GTPase (OPA1; antibody #5391, Cell Signaling, Danvers, MA, USA) and dynamin-related protein-1 (DRP1; antibody #80471, Cell Signaling) were assessed by western blot on mitochondrial protein extracts obtained by differential centrifugation, as mentioned. Total mitochondrial protein was measured by the Pierce Protein Assay method (Life Technologies, Carlsbad, CA, USA) in the same extract. Muscle cytokine profile and protein phosphorylation at insulin signalling mediators protein kinase-B (AKT^S473^), glycogen synthase kinase-3β (GSK3β^S9^), and ribosomal protein-S6 kinase (P70S6K^T421/S424^) were measured by high throughput xMAP technology (Magpix; Luminex Corporation, Austin, TX, USA), which, by using fluorescent coded reporter antibodies and microspheres, allows for multiple contemporary analyte immuno-mediated recognitions and discrete assays. In particular, samples were tested for a panel of proteins (Life Technologies, Carlsbad, CA, USA) using available commercial kits, which tested in two separate runs total and phosphorylated protein levels, according to the manufacturer’s instructions [12,19]. Briefly, frozen aliquots of gastrocnemius muscles were homogenized in a buffer (Tris 10 mM, pH 7.4, NaCl 100 mM, EDTA 1 mM, EGTA 1 mM, NaF 1 mM, Na_4_P_2_O_7_ 20 mM, Na_3_VO_4_ 2 mM, Triton X-100 1%, glycerol 10%, SDS 0.1%, deoxycholic acid 0.5%, PMSF 1 mM) added with protease inhibitors (P8340, Sigma-Aldrich, St. Louis, MO, USA). Following 20 min centrifugation (13,000× *g*, 4 °C), immuno-mediated analyte ibridation to magnetic beads, recognition and reporter quantification was performed as recommended by the manufacturer. Milliplex Analyst software 5.1 (Millipore, Billerica, MA, USA) was used for interpolating data to standard curve. Protein phosphorylation levels are expressed as phospho-protein units per total amount of the same protein in pg. Cytokine levels were also measured using xMAP technology with the relative commercial kits (Life Technologies, Carlsbad, CA, USA) and normalized by sample total protein content. Muscle proteolysis was assessed by western blot, as reflected by the validated marker of actin cleavage represented by the ratio of 14-kDa actin fragment over β-actin expression, as measured by densitometry [12,19].

**Ex vivo mitochondrial ROS generation**—ROS production in terms of H_2_O_2_ generation was measured in isolated intact mitochondria, isolated as mentioned, using the horseradish peroxidase-mediated Amplex Red (Invitrogen, Carlsbad, CA, USA) to resorufin conversion using a microplate fluorimeter (Infinite F200, Tecan Group, Männedorf, Switzerland, excitation wave length: 535 nm, emission: 595 nm, flashes: 25, integration time: 25 ms) during an incubation of mitochondria at 37 °C for 20 min with different respiratory substrates, as referenced and detailed in the figure legend [12,36,37]. Final substrate concentrations (mmol/L) were 8 glutamate, 4 malate (GM); 10 succinate (S); 4 glutamate, 2 malate, 10 succinate (GMS); 0.05 palmitoyl-L-carnitine, 2 malate (PCM). After further 20 min, state 3 respiration was obtained by the addition of ADP 1 mM. The integrity of mitochondrial function was checked by verifying for each preparation the effects of the uncoupling agent CCCP (5 µM) and antimycin A (1 μg/mL), as well as ADP (1 mM) on H_2_O_2_ production, since changes in mitochondrial activity after addition of these reagents are only possible in the presence of preserved mitochondrial function. As with ATP synthesis rate, results were normalized by citrate synthase activity, as assessed in the same mitochondrial preparation.

**Ex vivo superoxide anion production and redox state**—Superoxide anion production from gastrocnemius mitochondrial and non-mitochondrial sources was assessed ex vivo using the lucigenin chemiluminescent method in whole tissue homogenate [12,19,38]. Lucigenin concentration in the assay was 10 μmol/L, to prevent redox cycling. Briefly, freshly isolated tissue was cleaned of connective tissue, homogenised in ice cold buffer with protease inhibitors (Sigma, St. Louis, MO, USA), cleansed from debris by centrifugation (240× *g*, 15 min, 4 °C), and the obtained preparation tested in an array-type assay. Mitochondrial-specific superoxide production was measured considering the difference in light emission produced by the same homogenate when incubated with succinate (10 mM), a mitochondrial respiratory substrate, alone or with the addition of the mitochondrial uncoupling agent carbonyl cyanide chlorophenylhydrazone (5 mM). Similarly, for reduced nicotinamide adenine dinucleotide phosphate (NADPH) and xanthine oxidases derived superoxide, assessment was performed for each source by measuring light emission suppression induced by the addition of specific enzyme inhibitors in the presence of specific substrates (200 mM diphenyleneiodonium on 1 mM NADPH-stimulated production; 200 mM oxypurinol on 500 mM xanthine-induced generation for xanthine oxidase, respectively). In pilot experiments, the above concentrations did not alter luminescence in the absence of cellular homogenate. Results were normalized by sample protein content, measured as mentioned above. Gastrocnemius muscle measurements of total and oxidized glutathione (GSSG) were performed as described [37]. Briefly, ~50 mg of muscle sample was cleaned and homogenised in ice-cold 5% (wt/vol.) metaphosphoric acid (20 mL/g tissue). After clearance (12,000× *g*, 15 min, 4 °C) and appropriate dilution, samples were transferred to a 96-well plate and incubated in reaction buffer (containing DTNB 0.85 mM, glutathione reductase 10 U/mL). After the addition of NADPH 0.8 mM, the conversion of DTNB to TNB, which is proportional to the availability of GSH in the sample, was monitored on a spectrophotometer. Measurement of GSSG was performed with the same procedure, but after 1 h incubation of the diluted sample with 2-vynilpiridin 10% (*v*/*v*) and subsequent neutralization by addition of triethanolamine 16.6% (*v*/*v*), and calculating the reduced fraction by subtracting GSSG from total glutathione.

**Statistical analysis**—Sample size was decided by a priori power analysis as reported [14], assuming that expected differences in muscle mass would be comparable in magnitude to those previously observed in the same coronary artery ligation model. Unpaired ANOVA (GraphPad Prism 8.0, USA) was used for multiple group comparison and was followed by post hoc pairwise t-test with Bonferroni’s correction. For all tests, *p* < 0.05 was considered statistically significant. Data are presented as mean ± mean standard error.

## 3. Results

**Calorie Intake**, **Body Weight and Plasma Glucose**—The whole-study period average caloric intake was comparable in all groups, with no differences detected at any point between animals receiving PUFA-replaced vs. standard diet (Table 1). During the study period, physiologic increase in body weight was impaired in the CHF group compared to the sham-surgery group. The *n*-3 PUFA diet instead completely prevented impaired body weight gain in the CHF-PUFA compared to the CHF group. All groups shared comparable plasma glucose concentrations (Table 1).

**Figure 1:**
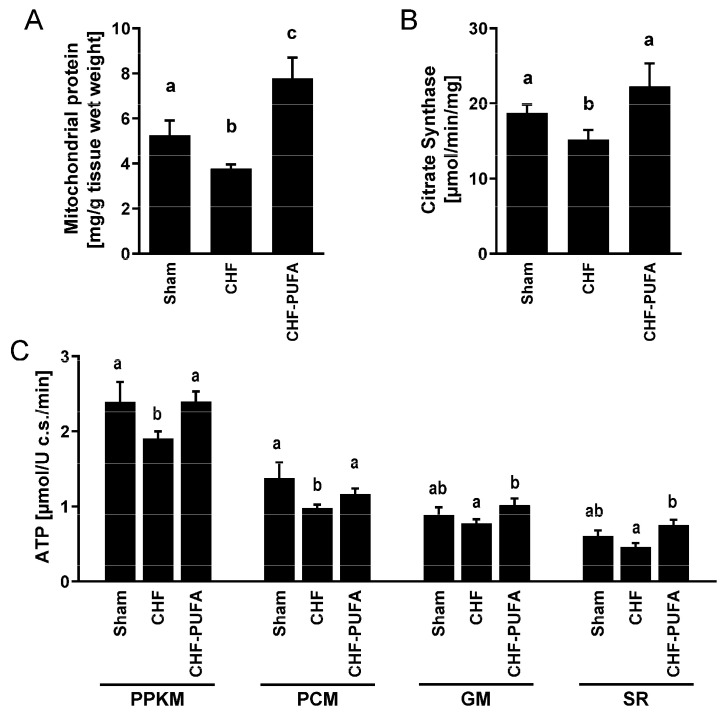
Muscle mitochondrial function. Impact of 8 week isocaloric, isolipidic *n*-3 PUFA-enriched diet in a mouse model after myocardial infarction-induced chronic heart failure (CHF) on left gastrocnemius (**A**) mitochondrial protein content, (**B**) citrate synthase activity, and (**C**) ATP synthesis rate in intact isolated mitochondria with different respiratory substrates (PPKM: Pyruvate+Palmitoyl-L-Carnintine+α-Ketoglutarate+Malate; PCM: Palmitoyl-L-Carnintine+Malate; GM: Glutamate+Malate; SR: Succinate+Rotenone) compared to chronic heart failure on standard diet (CHF) and to sham-operated animals (Sham). *p* < 0.05 among groups not sharing a letter, mean ± SEM, n = 8/group.

**Skeletal muscle mitochondrial function**, **dynamics regulators**, **ROS generation and redox state**—Compared to the sham-operated animals, CHF had lower muscle mitochondrial content, as assessed by mitochondrial protein measurement, as well as lower mitochondrial citrate synthase activity, a rate-limiting Krebs cycle enzyme. In agreement, both basal and ADP-stimulated ATP production were also lower in the skeletal muscle of CHF mice (Figure 1).

**Figure 2:**
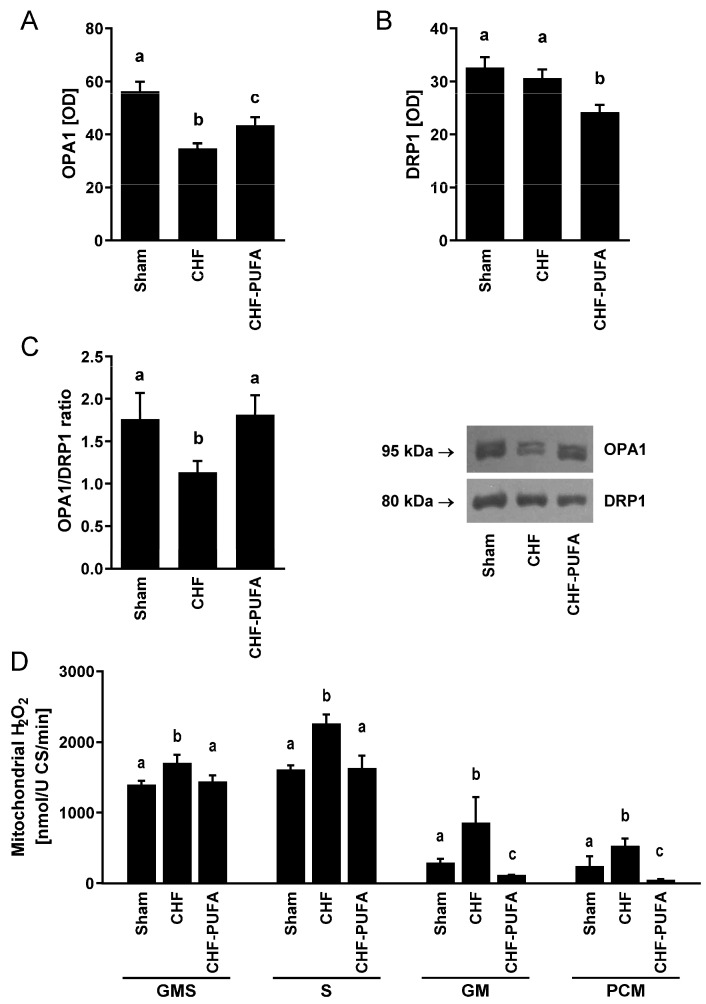
Muscle mitochondrial dynamics and mitophagy. Impact of 8 week isocaloric, isolipidic *n*-3 PUFA-enriched diet in a mouse model after myocardial infarction-induced chronic heart failure (CHF) on (**A**) OPA1 and (**B**) DRP1 protein levels; and (**C**) OPA1/DRP1 ratio, with representative blots, and on (**D**) muscle H_2_O_2_ production in intact isolated mitochondria with different respiratory substrates (GMS: 4 mmol/L glutamate, 2 mmol/L malate, 10 mmol/L succinate; S: 10 mmol/L succinate; GM: 8 mmol/L glutamate, 4 mmol/L malate; PCM: 50 μmol/L palmitoyl-L-carnitine, 2 mmol/L malate) compared to chronic heart failure on standard diet (CHF) and to sham-operated animals (Sham). *p* < 0.05 among groups not sharing a letter, mean ± SEM, n = 8/group.

**Figure 3:**
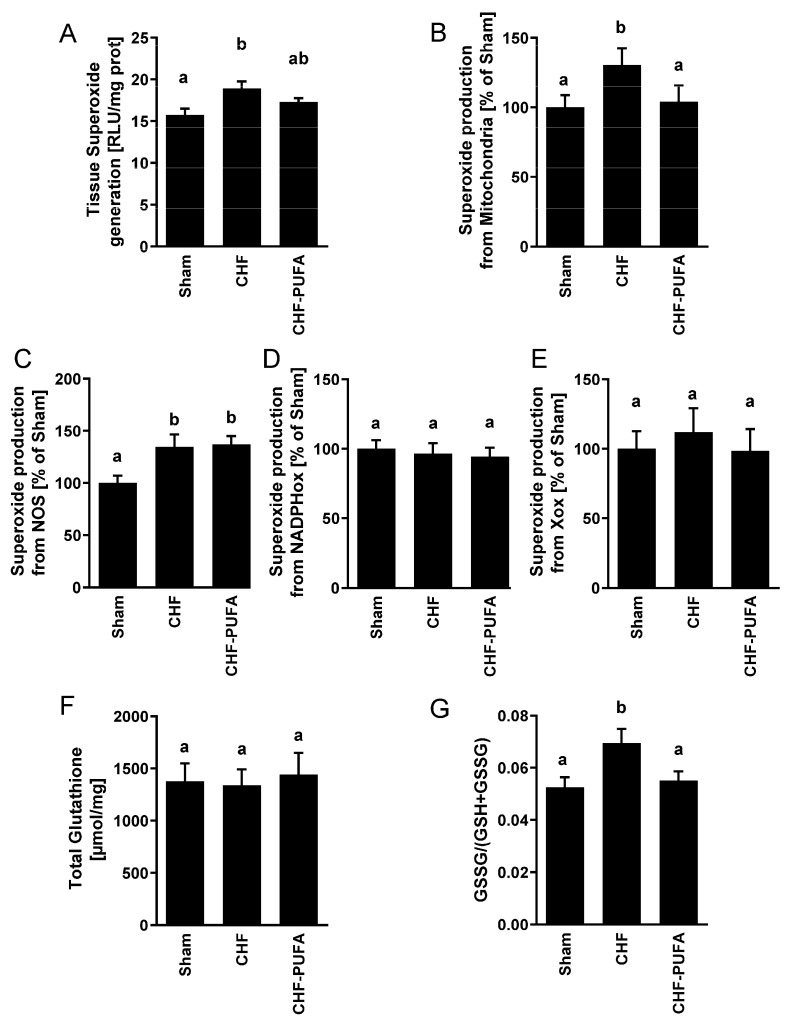
Muscle superoxide generation and redox state. Impact of 8 week isocaloric, isolipidic *n*-3 PUFA-enriched diet in a mouse model after myocardial infarction-induced chronic heart failure (CHF) on left gastrocnemius (**A**) total tissue, (**B**) mitochondria-, (**C**) uncoupled nitric oxide synthase (NOS)-, (**D**) Nicotinamide adenine dinucleotide phosphate oxidase (NADPHox)- and (**E**) Xanthine oxidase (XOx)-related superoxide generation, as well as on (**F**) total and (**G**) oxidated (GSSG) over total (GSSG+GSH) tissue glutathione compared to chronic heart failure on standard diet (CHF) and to sham-operated animals (Sham). *p* < 0.05 among groups not sharing a letter, mean ± SEM, n = 8/group.

Compared to Sham, the CHF group also had an altered gastrocnemius mitochondrial fission and fusion marker balance, with a lower ratio of the fusion OPA1 to fission DRP1 protein levels, mainly due to low OPA1 protein content with comparable DRP1 (Figure 2A–C), suggesting higher mitochondrial fragmentation. Specific superoxide generation and H_2_O_2_ emission from mitochondria were higher in CHF compared to Sham (Figure 2D). Also interestingly, superoxide production from additional sources, NADPH, and xanthine oxidase were not altered by CHF, whereas superoxide from NOS was enhanced in CHF compared to Sham. Clustered pro-oxidative derangements were further associated with a higher gastrocnemius GSSG-to-total glutatione ratio, a validated marker of redox balance whose elevation indicates a shift towards increased oxidative stress (Figure 3).

Importantly, isocaloric *n*-3 PUFA partial dietary lipid replacement completely recovered all CHF-induced mitochondrial and redox derangements, including dynamics regulators, mitochondrial ATP production, ROS emission, and GSSG-to-GSH+GSSG ratio, despite incomplete normalization of NOS superoxide overproduction (Figure 1, Figure 2 and Figure 3).

**Figure 4:**
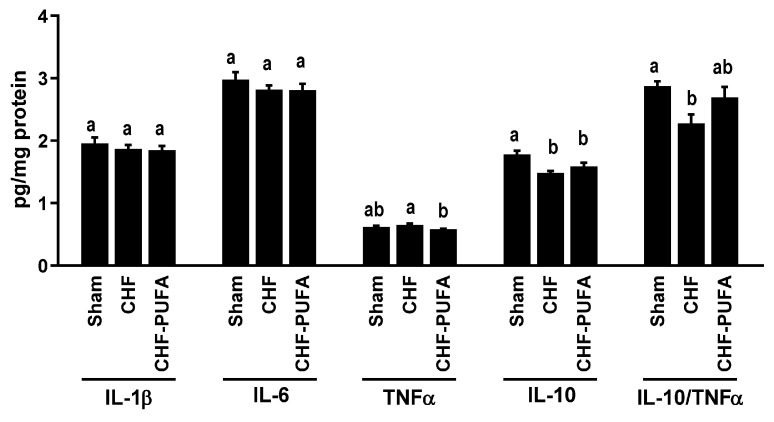
Muscle cytokine profile. Impact of 8 week isocaloric, isolipidic *n*-3 PUFA-enriched diet in a mouse model after myocardial infarction-induced chronic heart failure (CHF) on left gastrocnemius tissue protein levels of pro-inflammatory Interelukin (IL) 1β and 6 and Tumor Necrosis Factor α (TNFα), anti-inflammatory IL-10 and anti-inflammation index IL-10/TNFα ratio compared to chronic heart failure on standard diet (CHF) and to sham-operated animals (Sham). *p* < 0.05 among groups not sharing a letter, mean ± SEM, n = 8/group.

**Figure 5:**
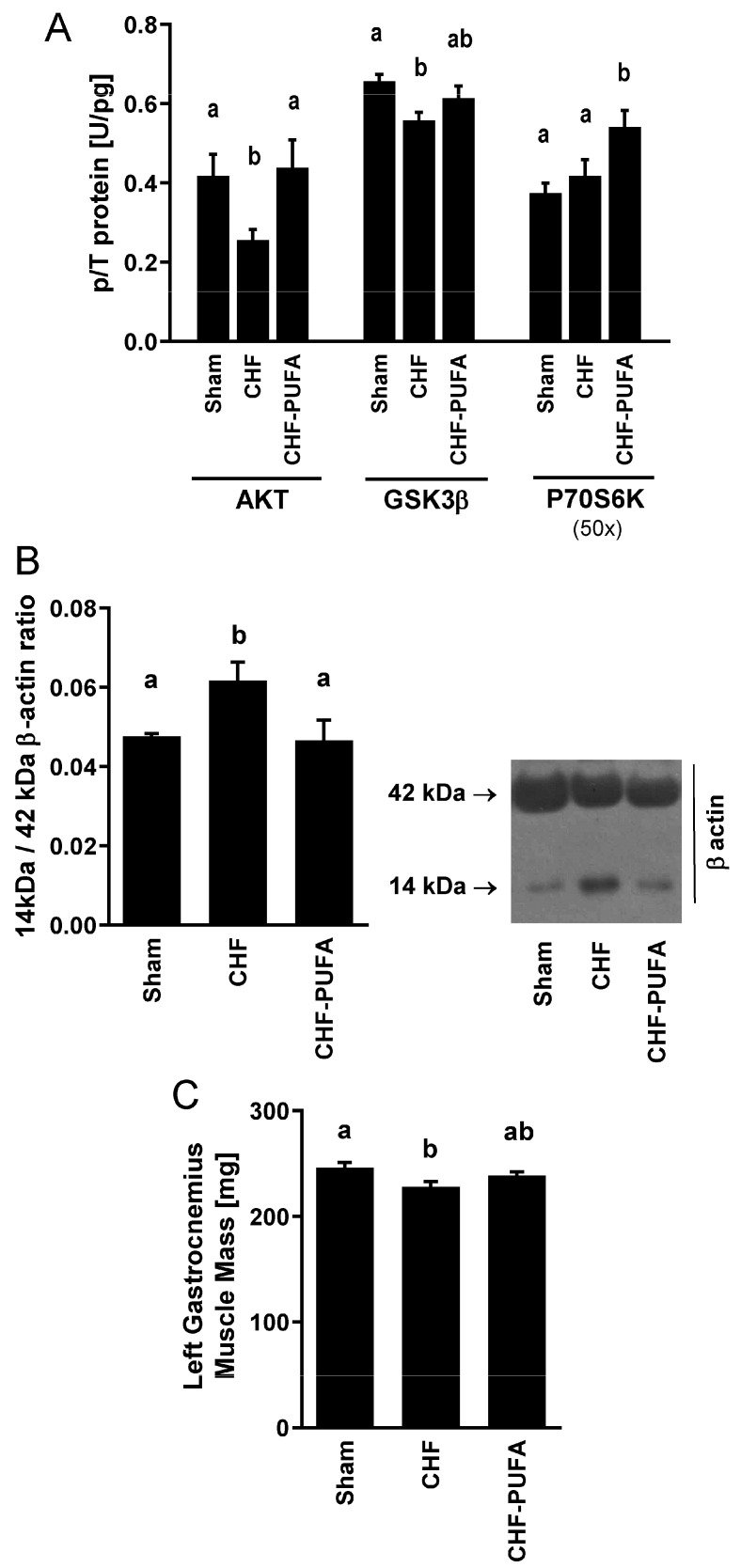
Muscle anabolic signalling activation, catabolism and mass. Impact of 8 week isocaloric, isolipidic *n*-3 PUFA-enriched diet in a mouse model after myocardial infarction-induced chronic heart failure (CHF) on left gastrocnemius (**A**) activation of insulin and anabolic signalling mediators in terms of relative phosphorylation (p/T) of protein kinase B (AKT^S473^), Glycogen synthase kinase 3β (GSK-3β^S9^) and Ribosomal protein S6 kinase (P70S6K^T421/S424^) to total protein ratio, (**B**) muscle 14 kDa actin fragment content, expressed as relative optical density over 42 kDa β-actin expression with representative blot and (**C**) muscle mass compared to chronic heart failure on standard diet (CHF) and to sham-operated animals (Sham). Results for P70S6K were scaled by multiplication for the indicated factor to improve figure readability. *p* < 0.05 among groups not sharing a letter, mean ± SEM, n = 8/group.

**Skeletal muscle inflammation**, **insulin signalling and muscle mass**—Compared to sham-operated animals, the pro-oxidative state observed in untreated CHF was associated with muscle pro-inflammatory changes in the IL-10-to-TNFα ratio, due to lower anti-inflammatory IL-10 levels (Figure 4). CHF also decreased activating phosphorylation of insulin signalling mediators involved in the upregulation of glucose uptake (pAKT^S473^ and pGSK3β^S9^; Figure 5A). Notably, CHF also inhibited insulin-dependent protein anabolic signalling (pP70S6K^T421/S424^) in gastrocnemius muscle (Figure 5A), directly supporting inhibition of protein synthesis mechanisms. This finding was also associated with higher muscle content of 14-kDa actin fragment, an established marker of protein breakdown [12,19,39] (Figure 5B). Consistent with the above observations, gastrocnemius muscle weight was lower in CHF than in S animals (Figure 5C). Conversely, *n*-3 PUFA completely recovered all CHF-induced alterations in cytokine levels, insulin signalling for glucose and protein metabolism, and muscle weight (Figure 4 and Figure 5).

## 4. Discussion

The current results show in a rodent CHF model that: (1) partial isocaloric replacement of dietary lipids with *n*-3 PUFA normalizes CHF-induced skeletal muscle mitochondrial derangements, including altered expression of mitochondrial dynamics regulators, low mitochondrial ATP production, and high mitochondrial ROS generation; (2) mitochondrial PUFA activities result in normalized tissue redox state, inflammation and insulin resistance, with normal protein catabolism markers and protection from muscle loss. These findings support novel roles of dietary *n*-3 PUFA to prevent deleterious muscle mitochondrial changes in CHF and muscle wasting, with a potential positive impact on patient clinical outcomes [40,41,42].

Skeletal muscle mitochondria are appealing treatment targets, since mitochondrial dysfunction and high ROS generation have been reported by us and others in CHF models [14,16] and patients [11,43], and may lead to tissue oxidative stress, inflammation, and insulin resistance with strong muscle-catabolic potential [9,10,12,14,43,44], as indeed confirmed in this study. The current results indeed demonstrate that isocaloric, isolipidic elevation of the *n*-3 PUFA dietary lipid fraction leads to complete normalization of CHF-induced mitochondrial changes. *n*-3 PUFA also normalized altered mitochondrial dynamics reflected by excess fission protein expression, potentially directly contributing to mitochondrial functional alterations in CHF, resulting in a normal fission-fusion protein ratio. The current data are also consistent with the major involvement of mitochondrial dysfunction in promoting muscle oxidative stress in CHF [11,16], as indicated by normalization by *n*-3 PUFA of oxidized-to-total glutathione, reflecting overall tissue redox balance, despite persistent increments of superoxide production from the NOS pathway, which deserves further direct investigation in future studies. These findings, therefore, support dietary *n*-3 PUFA utilization to prevent or treat muscle mitochondrial derangements and oxidative stress in CHF.

This study further investigated the impact of mitochondrial activities of *n*-3 PUFA on CHF-induced skeletal muscle catabolism. Selected muscle pro-inflammatory cytokine changes in CHF were observed, with no modification of pro-inflammatory cytokines but a low IL-10 to TNF-alpha ratio; this change was normalized by *n*-3 PUFA. *n*-3 PUFA also normalized CHF-impaired insulin signalling at the level of the key master-regulator of glucose and protein metabolism AKT and its substrate GSK3β. Also importantly, activation of the AKT-regulated downstream protein-anabolic regulator p70S6k [36] was also higher following *n*-3 PUFA dietary replacement [19]. Also consistent with enhanced insulin signalling, the validated marker of protein breakdown 14 kDa actin fragment was normalized by *n*-3 PUFA [12,19,39], whereas normal muscle mass directly confirmed the prevention of muscle catabolism. The above combined observations confirm the hypothesis that altered mitochondrial dynamics and pro-oxidative changes play a key role in CHF-induced muscle catabolism, and that *n*-3 PUFA-induced prevention of muscle wasting likely involves the prevention of mitochondrial derangements. The current findings, therefore, support a potential therapeutic role of dietary *n*-3 PUFA to preserve skeletal muscle mass in CHF.

Our recent findings of enhanced skeletal muscle mitochondrial oxidative stress associated with mitochondrial dysfunction and tissue catabolism in rodent CHF models with low ejection fraction [14,15] were notably fully confirmed in the current study. Indeed, loss of muscle mass is common in CHF patients, with a key potential negative impact on exercise capacity [4,5,43], fitness [4,7], and ultimately survival [7,8,41]. These results are also notably fully consistent with our recent report of muscle mitochondrial *n*-3 PUFA activities in experimental CKD [19]. Based on the current findings in a completely independent disease model, beneficial muscle mitochondrial activities of *n*-3 PUFA may hold therapeutic promise for different disease conditions characterized by skeletal muscle mitochondrial derangements. *n*-3 PUFA had previously been reported to reduce muscle ROS emission in some, although not all, available studies from aging and obesity models [24,30,45]. A recent study also supports *n*-3 PUFA activities to regulate mitochondrial dynamics in non-muscle tissues, with *n*-3 PUFA enhancing fusion protein levels [32]. Our recent findings in CKD [19] further suggested that mitochondrial activities could be at least partly mediated by enhanced expression of master-regulators of mitochondrial dynamics and biogenesis NRF2 and PGC1α, with activation of mitophagy, which might further contribute to improved mitochondrial function by disposal of damaged organelles. These additional mechanisms should be investigated in CHF in future studies.

Technical reasons prevented the assessment of muscle strength and exercise capacity in the current study. As mentioned above, low skeletal muscle function with impaired exercise capacity and strength are important determinants of CHF patient outcomes [1,3,5,18,41,42]. Higher exercise capacity and strength are, however, supported in this model by concomitant improvements of mitochondrial function and muscle mass. In addition, the preservation of skeletal muscle mass has independent clinical relevance, since loss of muscle mass is independently associated with negative outcomes in several disease conditions [40,46,47].

From a dietary, nutritional, and translational perspective, it should also be pointed out that our dietary approach completely prevented confounding changes in overall macronutrient balance and dietary intake. Modified macronutrient intake might have conversely contributed to inconsistent findings in previous studies, when *n*-3 PUFA were administered as dietary supplements [24,26,30,48,49]. The current *n*-3 PUFA diet included ~1% energy intake from EPA-DHA. The human equivalent intake would be ~2.5 g/day, i.e., the average dose in other major studies investigating *n*-3 PUFA in healthy humans (range 0.8–4.5 g/day) [50,51], indicating optimal potential clinical translation of the current results. Our study, therefore, supports the concept that nutritional treatment to enhance the *n*-3 PUFA dietary fraction in CHF patients, with unchanged calorie and macronutrient intake, has strong potential to prevent muscle mitochondrial and metabolic derangements, as well as muscle loss.

## 5. Conclusions

We conclude that *n*-3 PUFA isocaloric partial dietary lipid replacement normalizes skeletal muscle mitochondrial dynamics regulators, low mitochondrial ATP production and high mitochondrial ROS production in rodent chronic heart failure. Mitochondrial PUFA activities appear to further normalize tissue cytokine profile and insulin signalling, with the prevention of muscle wasting. Our results indicate that dietary *n*-3 PUFA may prevent muscle mitochondrial oxidative stress and protect from CHF-induced muscle catabolism and wasting, with a potential positive impact on patient morbidity and mortality.

## Figures and Tables

**Table 1:** **Animal characteristics.** Impact of 8 week isocaloric, isolipidic *n*-3 PUFA-enriched diet in a mouse model after myocardial infarction-induced chronic heart failure (CHF) on body weight, caloric intake and plasma glucose levels compared to standard diet and to sham-operated animals (Sham). Data timings refer to study start (T0: day 0), surgical CHF induction (T84: day 84), and sacrifice (T140: day 140). CHF-PUFA started feeding with PUFA-enriched diet after surgery. Other groups were fed with standard diet throughout the study. *p* < 0.05 between groups not sharing a letter for the same parameter, mean ± SEM, n = 8/group.

		Sham	CHF	CHF-PUFA
Body Weight [g]	T0	42.0 ± 1.5 ^a^	42.4 ± 0.9 ^a^	42.7 ± 1.2 ^a^
Body Weight [g]	T84	44.1 ± 1.2 ^a^	43.1 ± 1.3 ^a^	45.3 ± 1.6 ^a^
Body Weight [g]	T140	46.9 ± 1.3 ^a^	43.9 ± 1.1 ^b^	46.8 ± 2.5 ^ab^
Average daily caloric intake [kcal/d]	T0–T84	7.7 ± 0.6 ^a^	7.8 ± 0.3 ^a^	7.4 ± 0.8 ^a^
Average daily caloric intake [kcal/d]	T84–T140	7.7 ± 0.9 ^a^	8.1 ± 0.4 ^a^	8.8 ± 0.8 ^a^
Plasma Glucose [mg/dL]	T140	131 ± 4 ^a^	140 ± 3 ^a^	134 ± 5 ^a^

## Data Availability

Data may be provided upon reasonable request.
